# Myeloid response evaluated by noninvasive CT imaging predicts post-surgical survival and immune checkpoint therapy benefits in patients with hepatocellular carcinoma

**DOI:** 10.3389/fimmu.2024.1493735

**Published:** 2024-12-02

**Authors:** Kangqiang Peng, Xiao Zhang, Zhongliang Li, Yongchun Wang, Hong-Wei Sun, Wei Zhao, Jielin Pan, Xiao-Yang Zhang, Xiaoling Wu, Xiangrong Yu, Chong Wu, Yulan Weng, Xiaowen Lin, Dingjie Liu, Meixiao Zhan, Jing Xu, Limin Zheng, Yaojun Zhang, Ligong Lu

**Affiliations:** ^1^ State Key Laboratory of Oncology in South China, Guangdong Provincial Clinical Research Center for Cancer, Sun Yat-sen University Cancer Center, Guangzhou, China; ^2^ Guangdong Provincial Key Laboratory of Tumor Interventional Diagnosis and Treatment, Zhuhai Institute of Translational Medicine, Zhuhai People’s Hospital (Zhuhai Clinical Medical College), Jinan University, Zhuhai, China; ^3^ Medical AI Lab, Hebei Provincial Engineering Research Center for AI-Based Cancer Treatment Decision-Making, The First Hospital of Hebei Medical University, Shijiazhuang, China; ^4^ Department of Pathology, Xiangya Hospital, Central South University, Changsha, China; ^5^ Department of Management, School of Business, Macau University of Science and Technology, Macau, Macau SAR, China; ^6^ Department of Radiology, Zhuhai People’s Hospital, Jinan University, Zhuhai, China; ^7^ College of Medicine and Biological Information Engineering, Northeastern University, Shenyang, China; ^8^ Department of Radiology, The First Affiliated Hospital of Jinan University, Guangzhou, China; ^9^ Ministry of Education (MOE) Key Laboratory of Gene Function and Regulation, School of Life Sciences, Sun Yat-sen University, Guangzhou, China; ^10^ The Department of Cerebrovascular Disease, Zhuhai People’s Hospital, Jinan University, Zhuhai, China; ^11^ Guangzhou First People’s Hospital, The Second Affiliated Hospital, School of Medicine, South China University of Technology, Guangzhou, China

**Keywords:** hepatocellular carcinoma, radiomics, myeloid cells, prognosis, immunotherapy

## Abstract

**Background:**

The potential of preoperative CT in the assessment of myeloid immune response and its application in predicting prognosis and immune-checkpoint therapy outcomes in hepatocellular carcinoma (HCC) has not been explored.

**Methods:**

A total of 165 patients with pathological slides and multi-phase CT images were included to develop a radiomics signature for predicting the imaging-based myeloid response score (iMRS). Overall survival (OS) and recurrence-free survival (RFS) were assessed according to the iMRS risk group and validated in a surgical resection cohort (*n* = 98). The complementary advantage of iMRS incorporating significant clinicopathologic factors was investigated by the Cox proportional hazards analysis. Additionally, the iMRS in inferring the benefits of immune checkpoint therapy was explored in an immunotherapy cohort (*n* = 36).

**Results:**

We showed that AUCs of the optimal radiomics signature for iMRS were 0.941 [95% confidence interval (CI), 0.909–0.973] and 0.833 (0.798–0.868) in the training and test cohorts, respectively. High iMRS was associated with poor RFS and OS. The prognostic performance of the Clinical-iMRS nomogram was better than that of a single parameter (*p* < 0.05), with a 1-, 3-, and 5-year C-index for RFS of 0.729, 0.709, and 0.713 in the training, test, and surgical resection cohorts, respectively. A high iMRS score predicted a higher proportion of objective response (vs. progressive disease or stable disease; odds ratio, 2.311; 95% CI, 1.144–4.672; *p* = 0.020; AUC, 0.718) in patients treated with anti-PD-1 and PD-L1.

**Conclusions:**

iMRS may provide a promising method for predicting local myeloid immune responses in HCC patients, inferring postsurgical prognosis, and evaluating benefits of immune checkpoint therapy.

## Background

Hepatocellular carcinoma (HCC) is one of the leading causes of cancer-related death worldwide (1). Most HCCs arise from persistent inflammation, including hepatitis B or C virus (HBV or HCV) infections and nonalcoholic fatty liver disease (2). Therefore, immune cells constitute a highly complex and interactive milieu that contributes to the development and progression of HCC (3). Increasing evidence has also shown that tumor-infiltrating immune cells are potential prognostic and predictive factors for patient survival and therapeutic outcome (4–6). Substantial efforts have been made to depict the tumor microenvironment (TME) by integrating the information of local immune cells (7–10).

Myeloid cells are a population of heterogenous innate cells in the TME, including monocytes/macrophages, dendritic cells, neutrophils, and myeloid-derived suppressor cells (11). These cells are major components and critical regulators in the tumor contexture, which play a vital role in tumor initiation, progression, and therapy response (12, 13). Therefore, myeloid-based biomarkers have attracted particular attention to predict the prognosis and clinical benefit of patients (14–16). However, it usually needs to involve several myeloid markers due to the heterogenicity and plasticity of local myeloid cells in tumor tissues (8, 17), resulting in extra and redundant pathological examination. Previously, Wu et al. used 18 myeloid-related features to fit clinical data of patients with HCC and finally constructed a myeloid-specific prognostic signature (based on CD11b and CD169) named myeloid response score (MRS) (8). MRS reflects the changes in the myeloid response balance from antitumor to protumor activities and is closely related to the immune tolerance of CD8^+^ T cells. The findings indicate that MRS is accurate and useful in predicting post-surgery HCC prognosis and sorafenib efficacy for recurrent HCC. However, the small specimens cannot comprehensively capture the biological characteristics and reflect MRS levels in the whole tumor due to tumor heterogeneity and limitations in pathology specimen acquisition. Given the highly dynamic evolution and spatial heterogeneity of TME (18), a noninvasive, economical, and comprehensive panoramic view of the MRS assessment is still in great demand to decipher the tumor immune infiltrate.

Medical imaging, which allows noninvasive and comprehensive tumor evaluation, can reveal subtle relations between tumor texture and the molecular biological processes active in the TME (19). Multiphase dynamic enhancement CT scans can display lesions from multiple angles and directions, to fully reveal the blood supply and the intensification characteristics of patients with HCC. Unlike the visual interpretation of medical images, an emerging high-throughput computational method called “Radiomics” can reflect the molecular properties of tumor tissues through translating imaging data into high-dimensional quantitative data (20–22). It allows noninvasive, timely, and longitudinal evaluation of the entire tumor as well as its microenvironment to complement biopsies and tissue sections, demonstrating a great advantage in predicting vascular invasion, histological grade, prognosis, and therapeutic outcomes in HCC (23–28) and other tumors (29–32).

In view of the tumor immune microenvironment, the association and feasibility of radiomics-based biomarkers to tumor-infiltrating immune cell (33–36), the response of immunotherapy (37–41), and PD-L1/PD-L2 expression level (42–44) have also been investigated. Previously, Jiang et al. successfully developed a noninvasive radiomics-based predictor of ImmunoScore of gastric cancer from lymphoid and myeloid cells (45) and further validated its association with both disease-free survival and overall survival (OS) (35). The finding implied that it might be feasible to use radiomics to noninvasively predict MRS in HCC. Considering that only a fraction of patients benefit from the PD-1/PD-L1 monotherapy, it implies a great need to excavate a curative effect predictor for precise immunotherapy (46–48). In line with this, Yuming et al. (40) proposed a new imaging-based TME classifier, i.e., deep learning radiomics signature (DLRS), which could be used as a highly independent prognostic factor to accurately predict clinical response in patients treated with checkpoint blockade immunotherapy. The efficacy of predictive radiologic markers based on MRS in assessing the benefits of immune checkpoint therapy are unknown and need to be further explored in HCC.

In this study, we aimed to construct a predictive radiomics signature of MRS by combining triple-phase CT images and immunohistochemistry (IHC) staining from tumor biopsies for patients with HCC. A multi-phase CT (mp-CT) imaging-based predictor for MRS was validated to predict the local MRS, evaluate patient survival, and infer the benefits of immune checkpoint therapy in HCC.

## Methods

### Study design and patients

**Figure 1:**
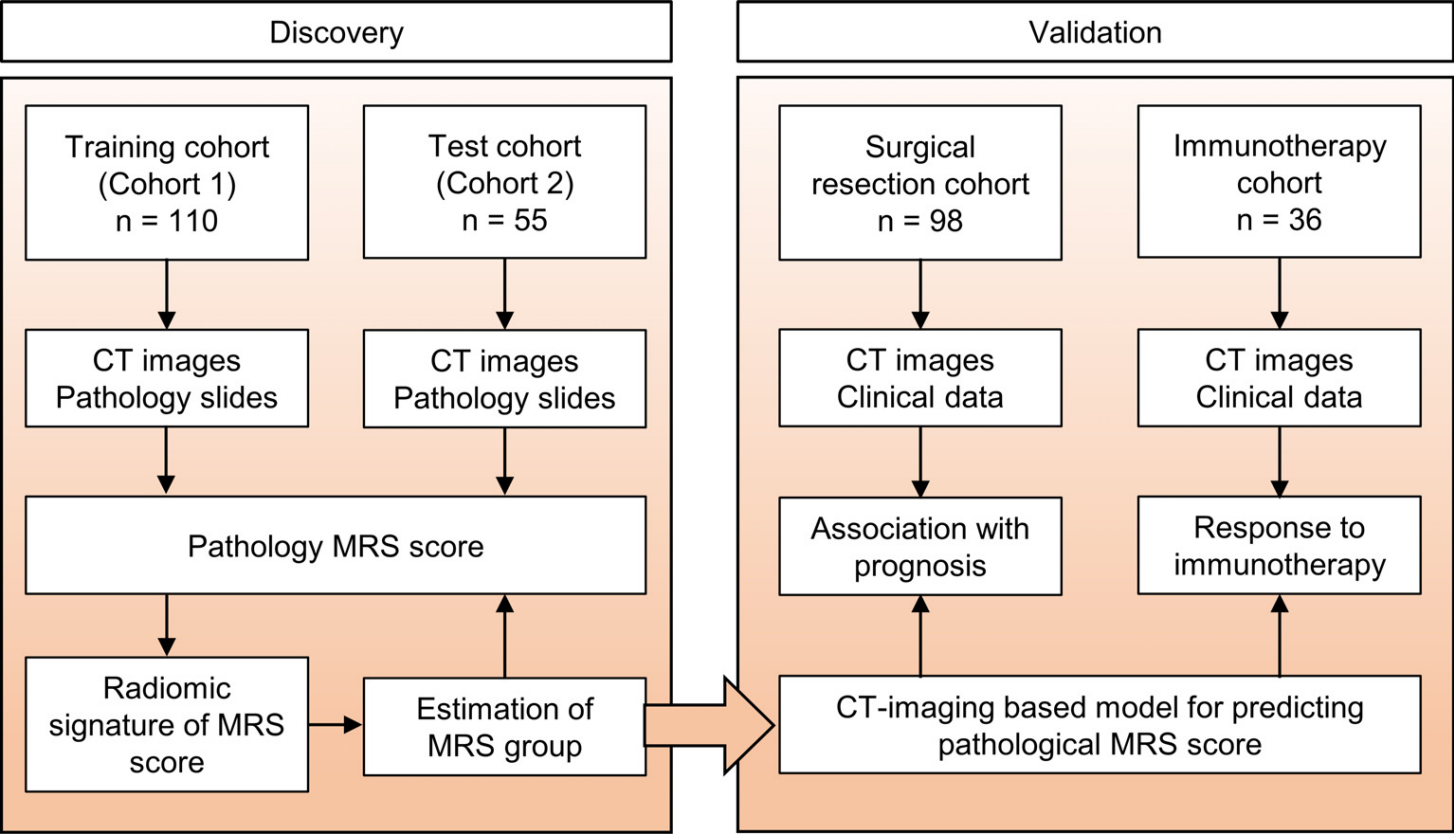
Study design. The training cohort and test cohort that contain data from patients with CT images and pathology slides were used to develop the radiomics signature of MRS. Two additional cohorts were used to validate the clinical and prognostic value of this radiomics signature. The surgical resection cohort comprised clinical data and the corresponding imaging data from patients with surgical resection. The immunotherapy cohort comprised advanced HCC patients who had been treated with anti-PD-1 or anti-PD-L1 therapy. MRS, myeloid response score.

The retrospective study was approved by the ethics committee of Sun Yat-sen University Cancer Center, and the requirement for informed consent was waived. The overall study design is shown in **Figure 1**. To develop the radiomics model for MRS, patients with HCC treated at our hospital were enrolled, in line with the inclusion and exclusion criteria. The inclusion criteria were as follows: (a) pathologic confirmation of HCC with available pathology slides and (b) preoperative contrast-enhanced liver CT performed. The exclusion criteria were as follows: (a) undergoing other treatments before surgery and (b) poor-quality radiologic or pathologic images. A total of 165 patients who performed presurgical liver CT scans and had available tumor samples for IHC staining from 2007 to 2010 were randomly split at a 7:3 ratio to cohort 1 (training cohort, *n* = 110) for the construction of the radiomics signature and cohort 2 (test cohort, *n* = 55) for performance evaluation. An additional surgical resection cohort (*n* = 98) including patients who were consecutively treated from 2010 to 2016 with CT scans and clinicopathological data was used for prognostic analysis based on the MRS radiomics signature. Moreover, another immunotherapy cohort (*n* = 36) with patients who underwent anti-PD-1/PD-L1 therapy from 2018 to 2020 was used to assess the outcome of immune checkpoint therapy in HCC. All the patients were followed up through telephone or admission notes to record recurrence and death. OS was calculated as the time from surgery to death or the last follow-up. Recurrence-free survival (RFS) was defined as the interval between the time of surgery to recurrence, the last follow-up for patients without recurrence, or death if no recurrence was observed.

### IHC staining and definition of MRS

Paraffin-embedded tumor tissues were cut into 4-μm sections, which were used for IHC staining. Tumor sections were sequentially deparaffinized and re-hydrated with xylene and a decreasing ethanol series. Subsequently, the slides were subjected to endogenous peroxidase activity elimination in 0.3% H_2_O_2_ for 10 min and heat-induced epitope retrieval in citrate buffer for 10 min. The sections were then incubated with anti-CD169 antibody (1:200, R&D Systems, Cat#AF5197) or anti-CD11b antibody (1:2000, Abcam, Cat#ab133357) overnight at 4°C. Diaminobenzidine (DAB) staining was performed with horseradish peroxidase-conjugated anti-rabbit/mouse Dako REAL™ EnVision™ detection systems (Dako, Cat# K5007) according to the manufacturer’s instructions. Brown color indicated positive signaling. All sections were counterstained with Mayer’s hematoxylin.

**Figure 2:**
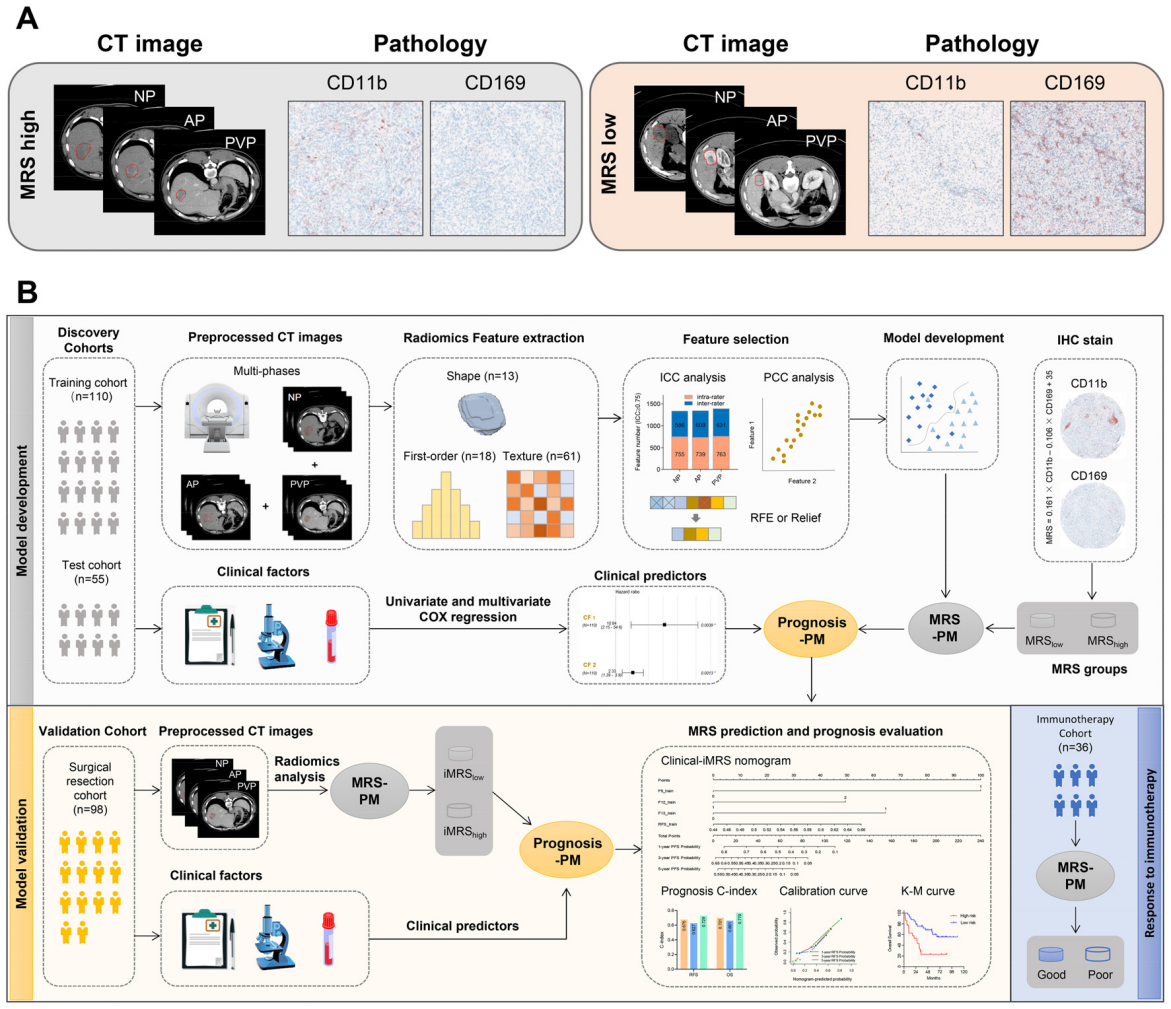
Radiomics workflow. **(A)** Representative imaging and pathology data of patients from MRS-high and MRS-low groups. **(B)** Radiomics workflow. MRS-PM represents the mp-CT radiomics signature with the best predictive performance for MRS. Prognosis-PM represents the prognostic Clinical-iMRS nomogram by combining iMRS and the significant clinicopathological features. NP, non-contrast phase; AP, arterial phase; PVP, portal venous phase; ICC, intraclass correlation coefficient; PCC, Pearson correlation coefficient; RFE, recursive feature elimination; iMRS, imaging-based MRS; mp-CT, multi-phase CT.

IHC-stained slides were scanned at ×20 magnification and evaluated by the Vectra 2.0 Automated Quantitative Pathology Imaging System and InForm Cell Analysis 2.2 (PerkinElmer) to count the numbers of CD169^+^ or CD11b^+^ cells (8). The density of cells was quantified as the number of positive cells per square millimeter area. The MRS was calculated as follows: MRS = 0.161 × CD11b − 0.106 × CD169 + 35 (0 ≤ MRS ≤ 100), to classify the HCC patients into two subgroups (MRS_low_, 0–60.6; and MRS_high_, 60.7–100; the cutoff was determined with the X-Tile software). IHC and CT images from two randomly selected patients in the MRS_low_ and MRS_high_ groups are shown in **Figure 2A**.

### CT acquisition, image preprocessing, and tumor segmentation

Triple-phase CT images of the liver, namely, non-contrast phase (NP), arterial phase (AP; 20–40 s postcontrast injection), and portal venous phase (PVP; 50–70 s postcontrast injection), were obtained after intravenous injection of contrast agent (Bayer Schering Pharma AG, Berlin, Germany; Ultravist Iopromide 370 mgI/mL) at a rate of 2–3 mL/s and a dose of 1–1.5 mL/kg bodyweight. The scanning range included the whole liver (from the diaphragmatic dome to the lower edge of the liver). Details regarding CT acquisition parameters are provided in **Supplementary Table S1.**

All the images were retrieved from the picture archiving and communication system. The gray values were normalized to a range of 0–255 to reduce scanner- and patient-dependent intensity variability in CT imaging. The manual segmentation containing the entire tumor for the NP, AP, and PVP images was independently conducted by two radiologists (readers 1 and 2 with 5- and 8-year experience in abdominal diagnosis, respectively) using the 3D Slicer software (version 5.0.3). Reader 2 repeated the segmentation a month later. A total of 50 patients were randomly selected from the MRS cohort to assess intra- and inter-segmentation reproducibility using dice similarity coefficient (DSC) and intraclass correlation coefficient (ICC). A feature with intra- and inter-ICC ≥ 0.75 was considered stable. A senior radiologist with 12 years of experience confirmed the segmentation variation. Any disagreement between the readers was discussed until a final consensus was reached.

### Radiomics feature extraction

Radiomics features were calculated in accordance with the guidelines of the Image Biomarker Standardization Initiative (https://theibsi.github.io/) (49). All voxels of the tumor region were first uniformly resampled to 1 × 1 × 1 mm^3^ with the cubic B-spline interpolation algorithm to improve the robustness of feature extraction. Then, a total of 962 radiomics features were extracted from each phase of CT images by using the Pyradiomics package (version 3.0.1), characterizing shape, intensity, and texture patterns of the tumor from the original and derived CT images (processed by the Laplacian of Gaussian or wavelet filter). The detailed extraction parameters and feature types are described in **Supplementary Method S1** and **Supplementary Table S2.**

### Radiomics feature selection and signature construction for MRS

Radiomics feature selection and signature construction to distinguish the MRS-high and -low group only used data from the training cohort. To eliminate the dimensions of feature magnitudes, *Z*-score normalization was applied. The Synthetic Minority Oversampling Technique (SMOTE) strategy was then used to remove the imbalance of the training cohort (40). In the procedure of feature selection, Pearson correlation coefficient (PCC) analysis was first conducted to obtain a feature subset with low redundancy; one of the features with PCC > 0.99 was randomly eliminated from further consideration. Recursive feature elimination (RFE) or Relief algorithm was further used for identifying the well-predictive features. Different classifiers, including random forest (RF), support vector machine (SVM), logistic regression (LR), K-nearest neighbor (KNN), and multilayer perceptron (MLP), were combined with five-fold cross-validation to obtain the optimal radiomics signature for each CT phase. A mp-CT radiomics signature was explored by fusing the predictive score of the triple-phase CT images. **Figure 2B** depicts the corresponding analysis flow.

### Imaging-based MRS construction for prognosis

Imaging-based MRS (iMRS) from mp-CT imaging was obtained from the predictive score of the optimal radiomics signature and then guided the risk stratification of MRS in subgroups. The cutoff value was defined by the optimal Youden’s index of predictive score within the training cohort and then applied to the other validation cohorts. Patients with a score higher than the cutoff value were classified as the high-risk group, and those with a score lower than the cutoff value were classified as the low-risk group. The prognostic value of iMRS risk groups in HCC was validated by Kaplan–Meier survival curves of RFS and OS. Additionally, we explored the advantage of the iMRS in inferring with the benefits of immune checkpoint therapy.

### Clinical and Clinical-iMRS score construction for prognosis

Clinicopathological features, including age, gender, hepatitis B surface antigen (HBsAg), alpha-fetoprotein (AFP), alanine aminotransferase (ALT), Child–Pugh class, tumor size, tumor number, vascular invasion, and Barcelona Clinic Liver Cancer (BCLC) stage, were enrolled. In the training cohort, significant variables associated with RFS (*p* < 0.05) in univariate Cox proportional hazards analysis were further adopted as covariates in a multivariate Cox proportional hazards analysis. The clinical prediction score was thus obtained by the output of the multivariate model to predict the status of RFS and OS. Considering clinical practice, we also analyzed the joint value of iMRS and the significant clinicopathological features in prognosis. A combined Clinical-iMRS nomogram was constructed by using the multivariate Cox proportional hazards analysis to calculate the Clinical-iMRS score.

### Statistical analysis

The difference in continuous variables between groups was analyzed by the Mann–Whitney test. Categorical variables were compared by the chi-square test or Fisher’s exact test, as appropriate. Radiomics feature extraction and signature development were performed using Python (version 3.7.6) and Pycharm (version 2020.1.5). Area under the receiver operator characteristic (ROC) curve (AUC), sensitivity, specificity, and accuracy were used to evaluate the predictive performance. We used the Delong test to compare the ROC curves of diverse radiomics signatures from the CT phases. For the survival analysis, the Kaplan–Meier curves for risk stratification were statistically tested by the log-rank test. The Harrell concordance index (C-index) was calculated for assessing the prognostic value of the scores. The calibration curve was plotted for the Clinical-iMRS nomogram. Note that a two-tailed *p* < 0.05 indicated statistical significance.

## Results

### Patient characteristics

The clinicopathological features of patients from different cohorts are summarized in **Table 1**. According to the MRS from IHC staining, 24.5% (27/110) and 23.6% (13/55) of patients were stratified into the MRS_high_ group in cohorts 1 and 2, respectively. Except for vascular invasion in cohort 2 (*p* = 0.014), there was no significant difference between the MRS_low_ and MRS_high_ groups (**Table 2**). The median follow-up duration was 49.0 months (range, 2–115 months) and 35.7 months (range, 2–107 months), respectively. The median OS and RFS for patients were 49.0 and 12.5 months in cohort 1, respectively. As for cohort 2, the median OS and RFS for patients were 35.7 and 11.7 months, respectively.

**Table 1:** Clinicopathological characteristics of patients with HCC included in four cohorts.

Characteristics	Cohort 1	Cohort 2	Surgical resection cohort	Immunotherapy cohort	*p*-value
No. of patients	110	55	98	36	
Age (years), median (range)	52 (13–74)	48 (22–76)	54 (20–87)	52 (31–70)	
Gender					
Female	15	4	13	6	0.523
Male	95	51	85	30	
HBsAg					
Negative	13	7	15	6	0.829
Positive	97	48	83	30	
AFP (ng/mL), median (range)	267.85 (0–121,000)	290.00 (0–121,000)	213.10 (1–121,000)	19,902.00 (4–121,000)	
ALT (U/L), median (range)	39.00 (0–118.0)	34.00 (8.0–140.0)	34.65 (11.0–389.3)	49.2 (19.6–254.7)	
Tumor size (cm), median (range)	6.75 (1.5–17.0)	7.0 (2.0–19.0)	5.0 (1.5–30.0)	11.2 (3.0–18.5)	
Child–Pugh class					
A	107	54	96	36	0.999
B–C	3	1	2	0	
Tumor number					
Single	74	40	80	N.A.	0.056
Multiple	36	15	18	N.A.	
Vascular invasion					
No	96	44	89	9	< 0.0001*
Yes	14	11	9	27	
BCLC stage					
0–A	59	33	43	0	< 0.0001*
B–C	51	22	55	36	

HCC, hepatocellular carcinoma; HBsAg, hepatitis B surface antigen; AFP, α-fetoprotein; ALT, alanine aminotransferase; BCLC, Barcelona Clinic Liver Cancer; N.A., not applicable.

*p*-values were analyzed by Fisher’s exact test.

**Table 2:** Clinicopathological characteristics of different MRS risk groups.

Characteristics	Cohort 1 (*n* = 110)		*p*-value	Cohort 2 (*n* = 55)		*p*-value
	MRS low	MRS high		MRS low	MRS high	
Age (years)						
≤48	33	13	0.504	20	9	0.215
>48	50	14		22	4	
Gender						
Female	13	2	0.351	4	0	0.562
Male	70	25		38	13	
HBsAg						
Negative	9	4	0.732	7	0	0.179
Positive	74	23		35	13	
AFP (ng/mL)						
≤25	24	10	0.476	13	2	0.477
>25	59	17		29	11	
ALT (U/L)						
≤40	44	14	1.000	30	8	0.511
>40	39	13		12	5	
Tumor size (cm)						
≤5	33	12	0.822	18	3	0.328
>5	50	15		24	10	
Child–Pugh class						
A	80	27	1.000	42	12	0.236
B–C	3	0		0	1	
Tumor number						
Single	56	19	1.000	31	9	0.734
Multiple	27	9		11	4	
Vascular invasion						
No	75	21	0.103	37	7	0.014*
Yes	8	6		5	6	
BCLC stage						
0–A	47	12	0.374	28	5	0.106
B–C	36	15		14	8	

*p*-values were analyzed by χ^2^ test or Fisher’s exact test, as appropriate.

HBsAg, hepatitis B surface antigen; AFP, α-fetoprotein; ALT, alanine aminotransferase; BCLC, Barcelona Clinic Liver Cancer. Data are presented as number of patients.

### Intra- and inter-segmentation reproducibility

The mean value and 95% confidence interval (CI) of DSC values [intra-rater DSC: 0.944 (0.936–0.953), 0.946 (0.940–0.953), and 0.946 (0.940–0.952); inter-rater DSC: 0.857 (0.838–0.876), 0.854 (0.842–0.865), and 0.858 (0.845–0.871)] for NP, AP, and PVP images were calculated, respectively. The results indicated that the difference in delineation was relatively small in this work. Furthermore, the median ICC values [intra-rater ICC: 0.967 (0.958–0.973), 0.981 (0.978–0.984), and 0.972 (0.966–0.976); inter-rater ICC: 0.854 (0.833–0.904), 0.915 (0.879–0.927), and 0.921 (0.902–0.928)] for NP, AP, and PVP images illustrated that the radiomics features also presented good reproducibility under the condition of small segmentation difference. 60.0% (577/962), 62.0% (596/962), and 64.3% (619/962) of the features from each phase were separately identified as good segmentation stability with ICC ≥ 0.75 of both intra- and inter-rater variability. The number of features under different ICC thresholds is presented in **Supplementary Table S3.**

### Predictive performance of the radiomics signatures for MRS

**Figure 3:**
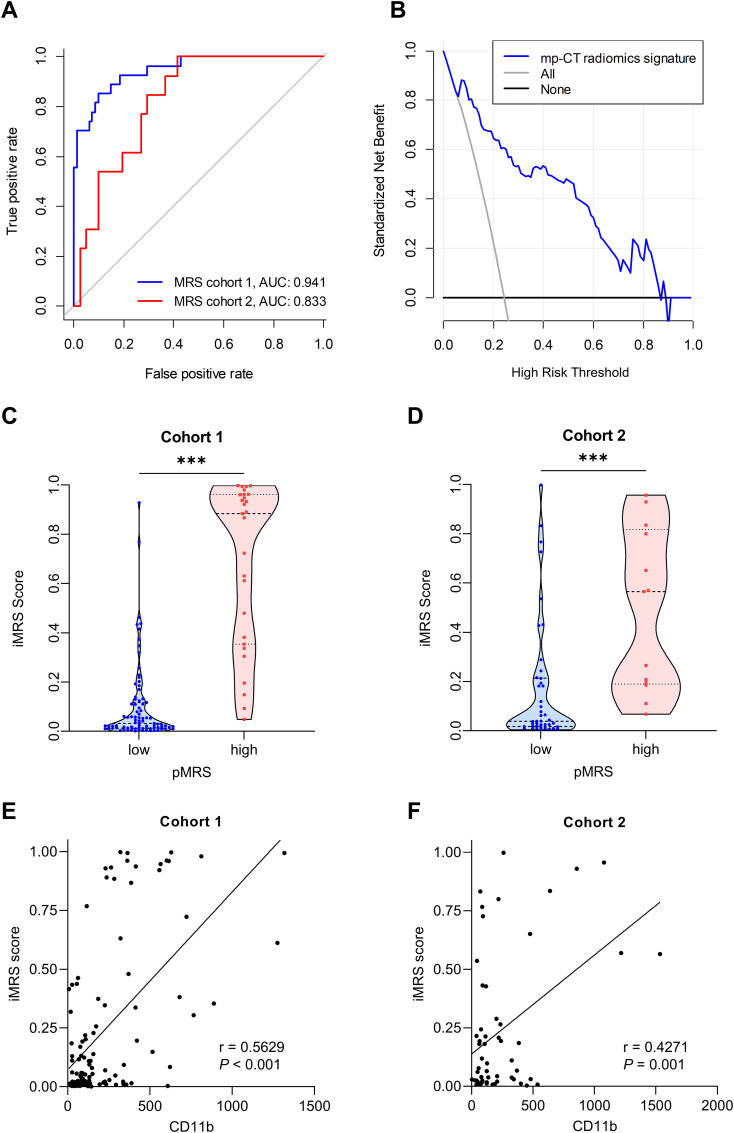
Performance of the radiomics signature. **(A)** ROC curves of the optimal mp-CT radiomics signature in cohorts 1 and 2. **(B)** Decision curve analysis. **(C, D)** iMRS of patients in pMRS-high or -low groups in cohort 1 **(C)** and cohort 2 **(D)**. Mann–Whitney test, ***p < 0.001. **(E, F)** The correlations between the number of tumor-infiltrating CD11b^+^ cells and iMRS in cohort 1 **(E)** and cohort 2 **(F)**. Spearman correlation analysis. ROC, receiver operator characteristic; pMRS, pathological MRS.

Two NP features (Relief and KNN), 8 AP features (RFE and MLP), and 11 PVP features (Relief and KNN) were identified as well-predictive through the combination optimization of different feature selection algorithms and classifiers, respectively (**Supplementary Table S4**). The optimization results of the applied machine learning algorithms are shown in **Supplementary Figure S1**. According to the AUC and accuracy (**Table 3**), the optimal radiomics signature from AP images (AUC, 0.769; 95% CI, 0.718–0.820) showed better performance compared with that from NP and PVP images in cohort 2. The corresponding sensitivity, specificity, and accuracy were 69.2%, 73.8%, and 72.7%, respectively. The predictive scores from the NP, AP, and PVP phase were further fused into the mp-CT radiomics signature, which demonstrated a pleasing improvement in performance with AUCs of 0.941 (95% CI, 0.909–0.973) and 0.833 (95% CI, 0.798–0.868) in cohorts 1 and 2, respectively (**Figure 3A**).

**Table 3:** Predictive performance of the optimal radiomics signatures identified from triple-phase CT images.

Datasets	Phase	AUC (95% CI)	*p*-value	Sensitivity (%)	Specificity (%)	Accuracy (%)
Cohort 1	NP	0.898 (0.863–0.932)	0.003*	100.0	63.9	72.7
	AP	0.735 (0.653–0.817)	<0.001*	51.9	83.1	75.5
	PVP	0.756 (0.690–0.822)	<0.001*	81.5	61.4	66.4
	mp-CT	0.941 (0.909–0.973)	Ref.	88.9	83.1	84.5
Cohort 2	NP	0.680 (0.631–0.730)	<0.001*	76.9	57.1	61.8
	AP	0.769 (0.718–0.820)	0.026*	69.2	73.8	72.7
	PVP	0.734 (0.686–0.783)	<0.001*	76.9	57.1	61.8
	mp-CT	0.833 (0.798–0.868)	Ref.	76.9	71.4	72.7

*p*-values were analyzed by the Delong test.

AUC, area under the curve; CI, confidence interval; NP, noncontrast phase; AP, arterial phase; PVP, portal venous phase; mp-CT, multiple-phase CT.

### Risk stratification from iMRS

Since the mp-CT radiomics signature provided the best predictive performance in both cohorts (all *p* < 0.05), iMRS was calculated and showed good net benefit for clinical use within the whole risk threshold range (**Figure 3B**). In both cohorts 1 and 2, the iMRSs of patients in the pathological MRS_high_ group were significantly higher than those in the pathological MRS_low_ group, (**Figures 3C, D**), which confirms the good discrimination of MRS groups based on iMRS. Moreover, the iMRSs were positively correlated with the number of tumor-infiltrating CD11b^+^ cells in both cohort 1 (*r* = 0.5629, *p* < 0.001; **Figure 3E**) and cohort 2 (*r* = 0.4271, *p* = 0.001; **Figure 3F**).

### Prognostic value of iMRS

**Figure 4:**
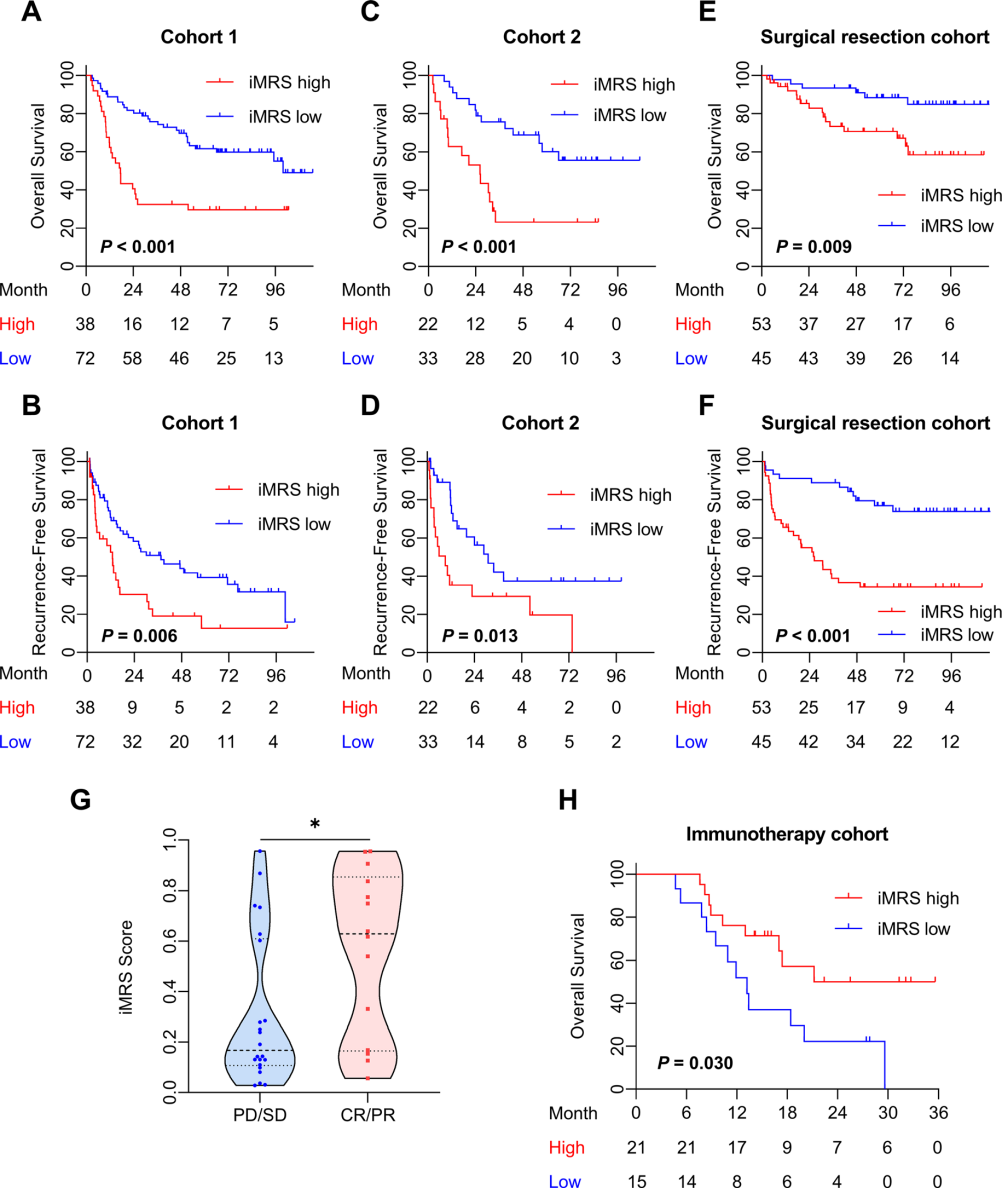
Prognostic and clinical value of the iMRS. **(A–F)** Overall survival and recurrence-free survival of patients relative to iMRS in cohort 1, cohort 2, and the surgical resection cohort. **(G)** iMRS of patients with complete CR/PR or PD/SD to anti-PD-1/PD-L1 therapy in the immunotherapy cohort. Mann–Whitney test, *p < 0.05. **(H)** Overall survival of patients relative to iMRS in immunotherapy cohort. CR, complete response; PR, partial response; PD, progressive disease; SD, stable disease.

The mp-CT radiomics score was calculated and patients were classified into high- and low-risk groups by iMRS with a cutoff value of 0.19. The Kaplan–Meier survival curves showed that patients with high iMRS had shorter OS and RFS in both cohorts 1 and 2 (**Figures 4A–D**). At the same time, the prognostic value of iMRS was confirmed in the surgical resection cohort. High iMRS indicated poorer OS (*p* = 0.009, **Figure 4E**) and RFS (*p* < 0.001, **Figure 4F**). The C-indexes of the iMRS were 0.627, 0.664, and 0.660 for predicting RFS in cohort 1, cohort 2, and the surgical resection cohort, respectively. Correspondingly, the C-indexes were 0.661, 0.649, and 0.648 for predicting OS.

The iMRS of patients in the complete response or partial response (CR/PR) group was significantly higher than those in the progressive disease or stable disease (PD/SD) group (*p* = 0.030, **Figure 4G**). High iMRS (odds ratio, 2.311; 95% CI, 1.144–4.672; *p* = 0.020) in the immunotherapy cohort predicted a higher proportion of CR/PR (vs. PD/SD; AUC, 0.718; 95% CI, 0.686–0.749) in patients who had received anti-PD-1 or anti-PD-L1 treatment. Patients with high iMRS had improved OS than those with low iMRS in the immunotherapy cohort (*p* = 0.030, **Figure 4H**).

### Prognostic value of clinical score

Clinical variables including Child–Pugh class, tumor number, vascular invasion, and BCLC stage were associated with RFS in univariate analysis (*p* < 0.001). The multivariate Cox model revealed that Child–Pugh class, tumor number, and vascular invasion were independent negative prognostic factors for RFS in HCC patients (**Supplementary Table S5**). We output the predicted probability as the clinical score. The prognostic C-indexes associated with RFS were 0.676, 0.654, and 0.610 for cohort 1, cohort 2, and the surgical resection cohort, respectively. The prognostic C-indexes associated with OS were 0.701, 0.647, and 0.667, respectively.

### Prognostic value of the Clinical-iMRS score

**Figure 5:**
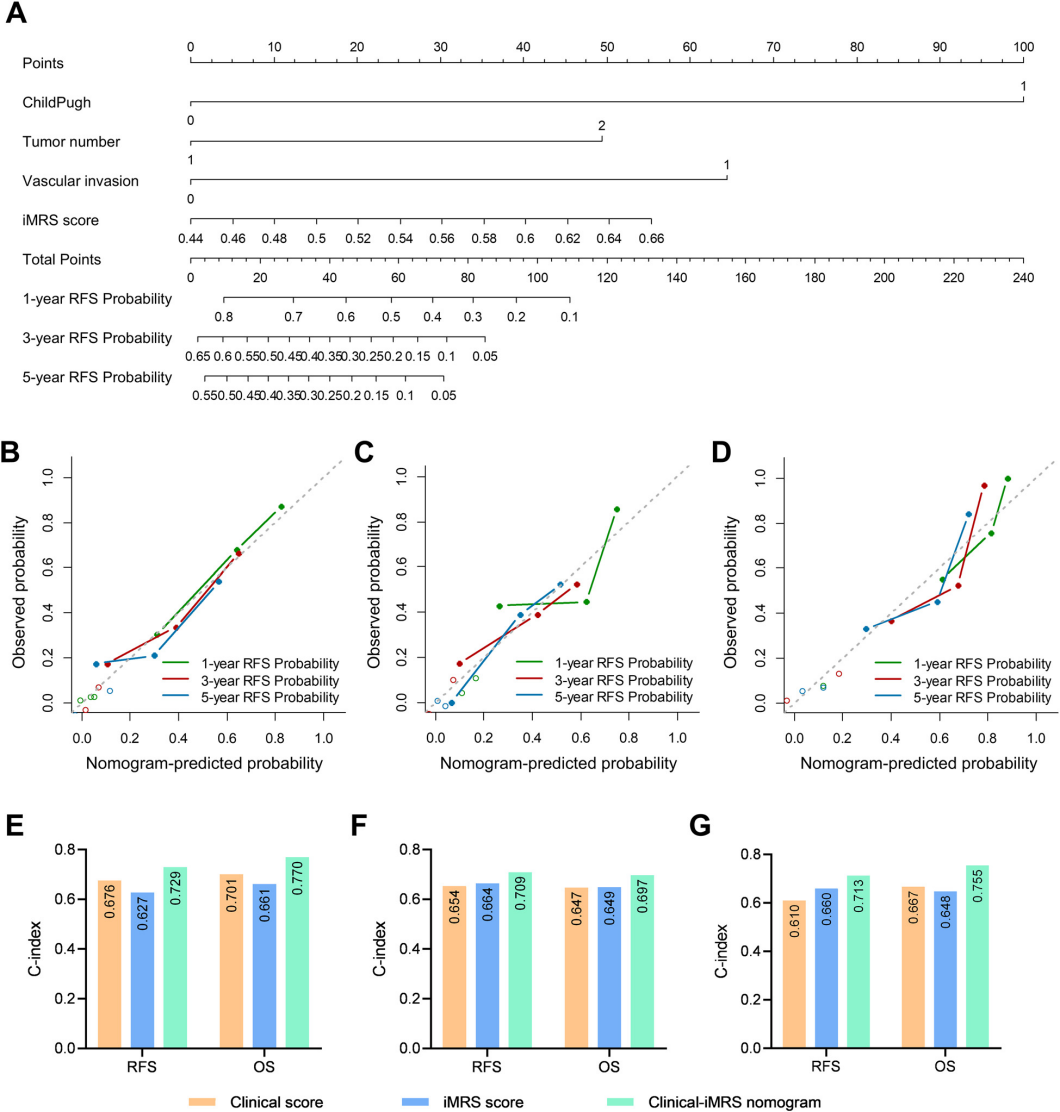
Clinical-iMRS nomogram and predictive performance evaluation. **(A)** The Clinical-iMRS nomogram to predict recurrence-free survival for post-surgical HCC patients. **(B–D)** Calibration curves for the nomogram in cohort 1 **(B)**, cohort 2 **(C)**, and the surgical resection cohort **(D)**. **(E–G)** C-index for assessing the prognostic value of the clinical score, iMRS, and combined Clinical-iMRS score in cohort 1 **(E)**, cohort 2 **(F)**, and the surgical resection cohort **(G)**. C-index, Harrell concordance index.

**Figure 6:**
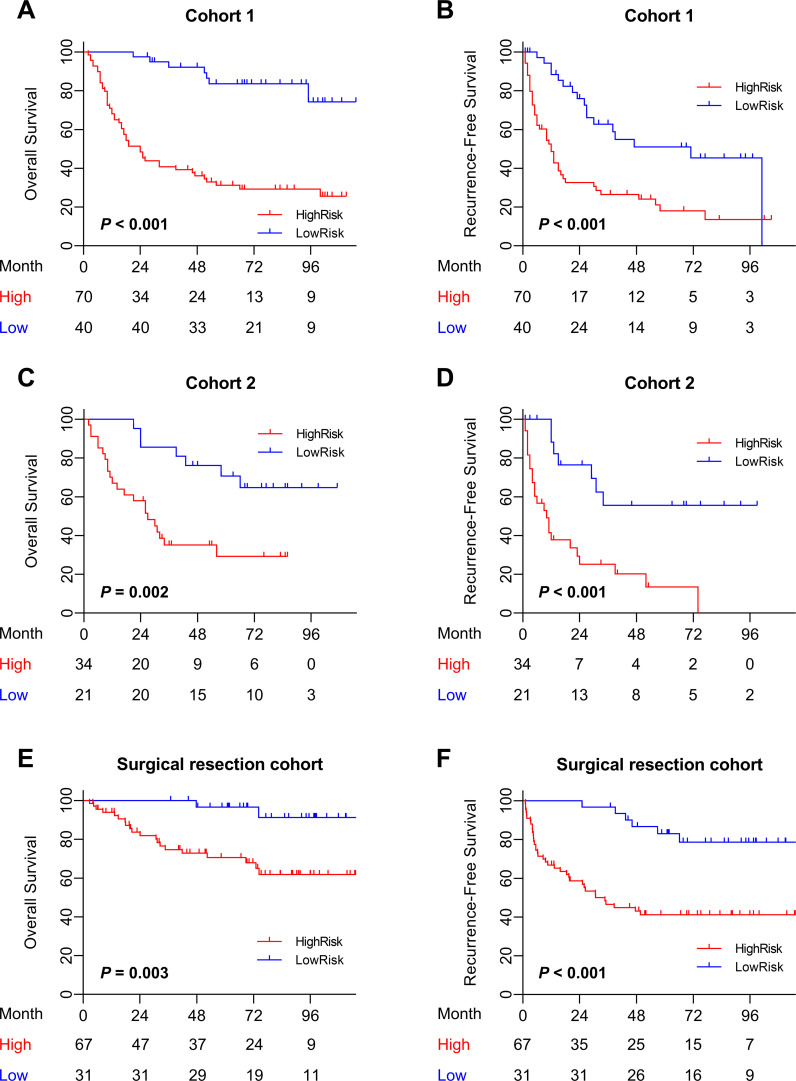
Prognostic value of the combined Clinical-iMRS model. **(A–F)** Overall survival and recurrence-free survival of patients relative to the Clinical-iMRS model (high risk or low risk) in cohort 1, cohort 2, and the surgical resection cohort.

Multivariate Cox analysis of clinicopathologic characteristics and iMRS for prognosis showed that high Child–Pugh class, tumor number, vascular invasion, and high iMRS were unfavorable predictors for RFS survival of HCC patients (**Table 4**), resulting in the Clinical-iMRS score. A well-discriminated and calibrated nomogram (**Figures 5A–D**) was subsequently developed, enhancing predictive accuracy compared to both iMRS and clinical scores. The C-indexes for 1-, 3-, and 5-year RFS prediction were 0.729, 0.709, and 0.713 for cohort 1, cohort 2, and the surgical resection cohort (**Figures 5E–G**), respectively. The advantage was also observed in OS with C-indexes of 0.770, 0.697, and 0.755, respectively. The Kaplan–Meier survival curves revealed that the nomogram showed notable capacity in predicting RFS and OS among postsurgical HCC patients (**Figure 6**).

**Table 4:** Univariate and multivariate Cox proportional hazards analysis of factors associated with recurrence.

Characteristics	Univariate Cox analysis		Multivariable Cox analysis	
	HR (95% CI)	*p*-value	HR (95% CI)	*p*-value
Age	1.005 (0.983–1.028)	0.678	–	–
Gender	1 (0.507–1.974)	0.999	–	–
HBsAg	1.503 (0.646–3.495)	0.344	–	–
AFP group	1.133 (0.664–1.932)	0.648	–	–
ALT group	1.017 (0.616–1.677)	0.948	–	–
**Child–Pugh class**	**16.040 (3.214–80.090)**	<0.001*	**10.375 (1.983–54.276)**	0.006*
Tumor size group	1.143 (0.685–1.909)	0.608	–	–
**Tumor number**	**2.607 (1.545–4.400)**	<0.001*	**3.183 (1.817–5.576)**	<0.001*
**Vascular invasion**	**3.487 (1.754–6.935)**	<0.001*	**4.519 (2.200–9.285)**	<0.001*
**BCLC stage**	**2.433 (1.465–4.042)**	<0.001*	–	–
**iMRS**	**2.311 (1.144–4.672)**	0.020*	**3.321 (1.579–6.986)**	0.002*

HBsAg, hepatitis B surface antigen; AFP, α-fetoprotein; ALT, alanine aminotransferase; BCLC, Barcelona Clinic Liver Cancer; HR, hazard ratio; CI, confidence interval. Characteristics with a *p* < 0.05 are displayed in bold.

## Discussion

Myeloid cells are a group of innate immune cells in the TME, which play a critical role in tumor initiation, progression, and therapy response in HCC. In this study, we sought to construct a CT-based radiomics model to predict MRS, to examine the prognostic value of iMRS, and to assess the association of iMRS with the outcome of patients treated with anti-PD-1 and anti-PD-L1 in HCC.

Compared with the commonly used TNM staging, various studies have shown that the type, density, and location of immune cells have superior prognostic value (50–52). Tumor-associated myeloid cells are important regulators and prognostic factors in tumor tissues. The myeloid-specific MRS derived from the CD169 and CD11b expression in HCC has been proven to be associated with the immune tolerance of CD8^+^ T cells, as well as the prognosis of post-surgery HCC (8). It is known that CD11b is often expressed by immunosuppressive myeloid cells in the blood and tumors, promoting tumor sensitivity to checkpoint blockade (53). This phenomenon has been confirmed in the latest study (54). As a specific marker, CD169 was useful in identifying suppressive tumor-associated macrophages in breast and endometrial cancers (55). Hence, MRS could provide a multidimensional tumor measure using various myeloid contextures. However, the evaluation of myeloid cells in tumor tissues usually requires IHC staining, which must be performed after surgery and specimen collection. The complexity and low efficiency of current methods limit the application of myeloid markers. Additionally, because of the heterogeneity and plasticity of myeloid cells in the TME, multiple antibodies are involved in the staining process, which can easily lead to statistical errors.

Our study successfully constructed a predictive model for MRS by an mp-CT radiomics signature, confirming the accuracy of iMRS and its noninvasive capacity for differentiating MRS in HCC. The derived iMRS was significantly higher in patients from the pathological MRS_high_ group and was positively correlated with the number of tumor-infiltrating CD11b^+^ cells. According to a previous study (56), image-derived textural diversity might possibly reflect increased immune cell infiltration, which increases tumor heterogeneity. Since the optimal mp-CT radiomics signature was constructed using multiple-phase CT images, the improved performance of the fusion model confirmed that the differences in gray levels across different phases could provide deeper supplementary information for evaluating the local myeloid response at the pre-surgery stage. In particular, the AUC of the radiomics signature from AP images was superior to that from NP and PVP images in cohort 2, which is consistent with the fact that arterial phase intensification is obvious and can present high signal characteristics (57). From the perspective of pathology, the outer layer of the tumor envelope in HCC patients presents relatively abundant compressed blood vessels and new bile ducts (58). The different blood supply of focal liver lesions is the basis of CT diagnosis and differential diagnosis. In general, the proposed method avoids the complex process of immunostaining and offers a noninvasive, efficient tool for calculating MRS.

Before the radiomics analysis of MRS, voxel resampling, gray-level discretization, and SMOTE strategy were adopted successively to reduce the data heterogeneity. SMOTE was considered the most prominent method for handling unbalanced data. As emphasized in previous studies (21), accurate segmentation of tumors remains a challenge affecting the popularization of radiomics models. Multiple segmentation by different radiologists can be used as a reliable method. Moreover, the inter- and intra-observer variability of manual segmentation and robustness of radiomics features were then investigated. The DSC and ICC values were higher than 0.85 in various segmentation scenarios, which demonstrated pleasing reproducibility in tumor delineation and made sure that our analysis was based on the accurate segmentation of tumor areas. From the perspective of features contained in the optimal signatures, we found that the features selected were all from the filtered CT images, and most of them were high-dimensional texture features. The data indicated that the processed images could amplify the differences between features, thereby providing more powerful texture information to distinguish the MRS groups.

The strength of our iMRS is that it shows good potential for predicting the survival of OS and RFS in patients with HCC as an independent prognostic biomarker. Kaplan–Meier survival curves reflected that there were significant differences between the two risk groups. Extensive studies have also reported on the similar association of tumor immunological infiltration with radiomics features and have accordingly verified the prognostic value of the noninvasive radiomics markers (33, 35, 36, 59, 60). Since the predictive C-indexes of iMRS for RFS and OS in different subsets were comparable to or worse than that of clinical scores, the Clinical-iMRS score significantly improved the predictive value for RFS. Our study revealed that the radiomics signature was an important supplementation to clinical features in survival analysis for HCC patients. Previous studies have found similar results (24, 25, 30, 34, 38, 61). It is an important trend for clinical utility to provide a comprehensive evaluation system using multiple factors that reflect the different biological properties of tumors.

Since the objective response rate of immunotherapy in most cancers is still relatively low (46–48), identifying reliable biomarkers from immune cells, such as the percentage of CD8^+^ cells and TLS structure, to quantify the TME associated with immunotherapy response, is still an emerging area of oncology research (33, 40, 62). In this study, a high iMRS predicted a higher proportion of objective response in patients treated with anti-PD-1 and PD-L1. Patients in the iMRS-high group had improved OS compared to those with low iMRS in the immunotherapy cohort. The results demonstrated that iMRS was significantly associated with the response of HCC patients to immune checkpoint therapy, which could identify patients most likely to benefit from anti-PD-1 and PD-L1 therapy. In mechanism, the MRS was useful to describe the immune microenvironment of HCC, and HCC patients with high MRS displayed immunosuppressive TMEs (8). On the one hand, MRS_high_ tumors are associated with CD8^+^ T-cell exhaustion, especially PD-1 expression on infiltrating T cells. On the other hand, the elevation of PD-L1 expression on Mφs and tumor cells is significantly associated with high MRS. Therefore, we suggest that noninvasive iMRS can help promote the surveillance of the TME and the translational application of immunotherapy effectively and rationally.

Our study also has several limitations. First, the data were retrospectively collected from a single center, and the amount of data was small. It is important to note that we grouped the HCC patients over different time periods to provide a longitudinal time design to verify the generalization performance of the model. This also compensates for the shortcomings of a single center. Of course, more data from other centers will be collected in the future to further prospectively validate our findings. Second, despite our efforts to select relatively stable radiomics features through intra- and inter-ICC analysis, an automated and accurate segmentation strategy is necessary and can improve the efficiency of radiomics analysis. Third, while the radiomics signature was associated with CD11b^+^ cells’ infiltration, the overview of the immune infiltration pattern from single-cell RNA sequencing could better explain the biological meaning of radiomics patterns of our iMRS. Lastly, multiomics fusion holds great potential for future development, and we hope to combine other multidimensional data with iMRS to better characterize the TME of patients with HCC and assist clinical treatment decision-making (63).

## Conclusions

Our study constructed an accurate and efficient predictive model for MRS, confirmed the correlation of the radiomics signature and tumor-infiltrating myeloid cells, further revealed the prognostic value of iMRS for survival, and assessed the association of iMRS with the outcome of anti-PD-1 and PD-L1 therapy in HCC. Our study provided a promising and noninvasive tool to evaluate the TME and to assist immunotherapeutic decisions in clinical trials, thus enabling a more tailored therapeutic approach with improved outcomes for HCC patients.

## Data Availability

The original contributions presented in the study are included in the article/**Supplementary Material**. Further inquiries can be directed to the corresponding authors.
