# Supplementary material for: Myeloid response evaluated by noninvasive CT imaging predicts post-surgical survival and immune checkpoint therapy benefits in patients with hepatocellular carcinoma
Source: Front Immunol. 2024 Dec 2;15:1493735. doi: 10.3389/fimmu.2024.1493735 (PMC11646988; doi:10.3389/fimmu.2024.1493735)
Supplement: Supplementary file 1 [file DataSheet1.pdf]

Supplementary Materials

Supplementary Method S1: Radiomics feature extraction

In this study, original and filtered CT images were used for radiomics feature extraction using the open-source Pyradiomics package(1) in Python 3.7.6. According to the YAML-convention (www.yaml.org), three types of parameters for radiomics feature extraction were defined as follows:

i) Image types: ‘Original’, ‘Laplacian of Gaussian (LoG)’, and ‘Wavelet’. For the derived images, LoG filter was used for image filtration with sigma of 2 mm, 4 mm, and 6 mm, respectively. Wavelet filter was applied to focus features on the different decomposition and approximation level of the original contoured volumes, and the bin width of which was set as 10.

ii) Hyperparameter setting: ‘geometryTolerance’, ‘binWidth’, ‘interpolator’, and ‘resampledPixelSpacing’. The geometry tolerance was set as 1.00000e-4. Voxels in each volume were resampled to a unified voxel size of 1×1×1 mm^3 using ‘sitkBSpline’ while the bin width was set as 25.

iii) Feature groups: ‘shape’, ‘firstorder’, ‘glcm’, ‘glrlm’, ‘glszm’, and ‘ngtdm’.

The radiomics feature group and name are listed in supplementary Table S2. The detailed descriptions and mathematical formulas can be referred to https://pyradiomics.readthedocs.io.

References

1. Joost J M vG, Andriy F, Chintan P, Ahmed H, Nicole A, Vivek N, et al. Computational Radiomics System to Decode the Radiographic Phenotype. *Cancer Res*. (2017) 77. doi: 10.1158/0008-5472.Can-17-0339.

Supplementary Table S1. CT acquisition parameters

**CT scanner**	Spectral CT (GE Discovery CT 750HD, GE Healthcare)	128-slice spiral CT (Aquilion TSX-101A, Toshiba)	256-slice spiral CT (Brilliance 16, Philips)
Tube voltage (kV)	120	120	120
Tube current (mA)	300	250/300	234/281/313/350/375
Matrix size	512×512	512×512	512×512
Section thickness (mm)	5	5	5
Section interval (mm)	5	5	5
Helical pitch (kV)	0.984	1.0	1.0

Supplementary Table S2. Types of the extracted radiomics features

Feature group	Number	Feature name	Feature name
Shape	13	Flatness	Mesh volume
		Least axis length	Minor axis length
		Major axis length	Sphericity
		Maximum 2D diameter (Column)	Surface area
		Maximum 2D diameter (Row)	Surface area to volume ratio
		Maximum 2D diameter (Slice)	Voxel volume
		Maximum 3D Diameter	
First-order	18	10th percentile	Median
		90th percentile	Minimum
		Energy	Range
		Entropy	Robust mean absolute deviation
		Interquartile range	Root mean squared
		Kurtosis	Skewness
		Maximum	Total Energy
		Mean absolute deviation	Uniformity
		Mean	Variance
GLCM		Auto correlation	Inverse difference normalized
		Cluster prominence	Informational measure of correlation1
		Cluster shade	Informational measure of correlation2
		Cluster tendency	Inverse variance
		Contrast	Joint average
	24	Correlation	Joint energy
		Difference average	Joint entropy
		Difference entropy	Maximal correlation coefficient
		Difference variance	Maximum probability
		Inverse difference	Sum average
		Inverse difference moment	Sum entropy
		Inverse difference moment Normalized	Sum squares
GLRLM	16	Gray level non-uniformity	Run entropy
		Gray level non-uniformity Normalized	Run length non-uniformity
		Gray level variance	Run length non-uniformity Normalized

		High gray level run emphasis	Run percentage
		Long run emphasis	Run variance
		Long run high gray level Emphasis	Short run emphasis
		Long run low gray level Emphasis	Short run high gray level Emphasis
		Low gray level run emphasis	Short run low gray level Emphasis
GLSZM	16	Gray level non-uniformity	Size-zone non-uniformity
		Gray level non-uniformity Normalized	Size-zone non-uniformity Normalized
		Gray level variance	Small area emphasis
		High gray level zone emphasis	Small area high gray level Emphasis
		Large area emphasis	Small area low gray level Emphasis
		Large area high gray level Emphasis	Zone entropy
		Large area low gray level Emphasis	Zone percentage
		Low gray level zone emphasis	Zone variance
NGTDM	5	Busyness	Contrast
		Coarseness	Strength
		Complexity	

Abbreviations: GLCM, gray level co-occurrence matrix; GLRLM, gray level run length matrix; GLSZM, gray level size zone matrix; NGTDM, neighboring gray tone difference matrix.

Supplementary Table S3. Number of stable radiomics features under different intra- and inter-rater ICC thresholds.

ICC	NP		AP		PVP	
	Intra-rater	Inter-rater	Intra-rater	Inter-rater	Intra-rater	Inter-rater
≥ 0.75	755	586	739	609	763	631
≥ 0.80	725	555	718	575	741	609
≥ 0.85	688	482	683	536	711	571
≥ 0.90	638	452	648	499	665	515

Abbreviations: ICC, intraclass correlation coefficient; NP, noncontrast phase; AP, arterial phase; PVP, portal venous phase.

Supplementary Table S4. Radiomics features included in the optimal radiomics signatures identified from triple phase CT images

Phase	Radiomics features
NP	wavelet-LLH_glszm_ZonePercentage
	log-sigma-2-0-mm-3D_ngtdm_Strength
AP	log-sigma-2-0-mm-3D_firstorder_Variance
	log-sigma-2-0-mm-3D_glcm_Autocorrelation
	log-sigma-4-0-mm-3D_ngtdm_Strength
	log-sigma-6-0-mm-3D_glszm_GrayLevelNonUniformity
	wavelet-LLH_glszm_SmallAreaHighGrayLevelEmphasis
	wavelet-LHL_glszm_SmallAreaEmphasis
	wavelet-HLH_glcm_DifferenceEntropy
	wavelet-HHL_glszm_GrayLevelNonUniformityNormalized
PVP	wavelet-HHH_glszm_SmallAreaHighGrayLevelEmphasis
	log-sigma-4-0-mm-3D_firstorder_Kurtosis
	wavelet-LHH_glszm_GrayLevelNonUniformityNormalized
	wavelet-HHH_glszm_HighGrayLevelZoneEmphasis
	wavelet-LHL_firstorder_Median
	wavelet-LLH_glszm_HighGrayLevelZoneEmphasis
	log-sigma-6-0-mm-3D_ngtdm_Busyness
	log-sigma-6-0-mm-3D_firstorder_Kurtosis
	wavelet-HHH_glszm_GrayLevelNonUniformityNormalized
	wavelet-LHH_glrlm_GrayLevelNonUniformityNormalized
	wavelet-LHL_glszm_SmallAreaLowGrayLevelEmphasis

Abbreviations: NP, non-enhanced phase; AP, arterial phase; PVP, portal venous phase.

Supplementary Table S5. Multivariate COX proportional hazards analysis of clinicopathological associated with recurrence

Characteristics	Multivariate COX	
	HR (95% CI)	*P* value
Child-Pugh class	8.573 (1.647-44.622)	0.011*
Tumor number	2.796 (1.613-4.844)	<0.001*
Vascular invasion	4.232 (2.078-8.617)	<0.001*

Abbreviations: HR, hazard ratio; CI, confidence interval.

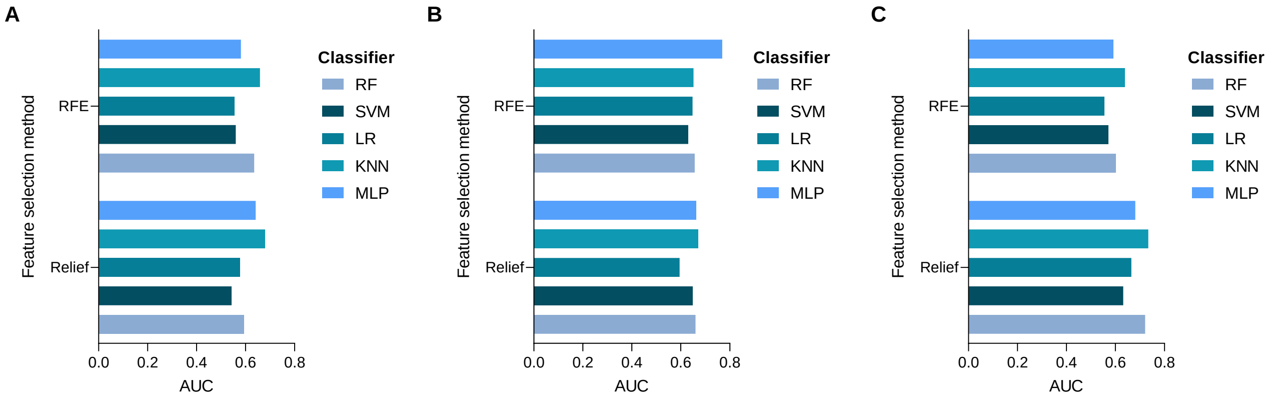

Supplementary Figure S1. Performance comparisons of different feature selection algorithms and classifiers in the test cohort. (**A**) NP; (**B**) AP; (**C**) PVP. **Abbreviations:** NP, noncontrast phase; AP, arterial phase; PVP, portal venous phase; RF, random forest; SVM, support vector machine; LR, logistic regression; KNN, K-nearest neighbor; MLP, multilayer perceptron; RFE, recursive feature elimination.
